# Knowledge-based compact disease models identify new molecular players contributing to early-stage Alzheimer’s disease

**DOI:** 10.1186/1752-0509-7-121

**Published:** 2013-11-07

**Authors:** Anatoly Mayburd, Ancha Baranova

**Affiliations:** 1 The Center of the Study of Chronic Metabolic Diseases, School of Systems Biology, College of Science, George Mason University, Fairfax, VA 22030, USA; 2 Research Centre for Medical Genetics, RAMS, Moskvorechie 1, Moscow, Russia

**Keywords:** Signature, Network, Knowledge-based algorithms, Alzheimer’s, Protein traffic vesicles, Affymetrix, Illumina, Antihypertensive drugs

## Abstract

**Background:**

High-throughput profiling of human tissues typically yield as results the gene lists comprised of a mix of relevant molecular entities with multiple false positives that obstruct the translation of such results into mechanistic hypotheses. From general probabilistic considerations, gene lists distilled for the mechanistically relevant components can be far more useful for subsequent experimental design or data interpretation.

**Results:**

The input candidate gene lists were processed into different tiers of evidence consistency established by enrichment analysis across subsets of the same experiments and across different experiments and platforms. The cut-offs were established empirically through ontological and semantic enrichment; resultant shortened gene list was re-expanded by Ingenuity Pathway Assistant tool. The resulting sub-networks provided the basis for generating mechanistic hypotheses that were partially validated by literature search. This approach differs from previous consistency-based studies in that the cut-off on the Receiver Operating Characteristic of the true-false separation process is optimized by flexible selection of the consistency building procedure. The gene list distilled by this analytic technique and its network representation were termed Compact Disease Model (CDM). Here we present the CDM signature for the study of early-stage Alzheimer’s disease. The integrated analysis of this gene signature allowed us to identify the protein traffic vesicles as prominent players in the pathogenesis of Alzheimer’s. Considering the distances and complexity of protein trafficking in neurons, it is plausible that spontaneous protein misfolding along with a shortage of growth stimulation result in neurodegeneration. Several potentially overlapping scenarios of early-stage Alzheimer pathogenesis have been discussed, with an emphasis on the protective effects of AT-1 mediated antihypertensive response on cytoskeleton remodeling, along with neuronal activation of oncogenes, luteinizing hormone signaling and insulin-related growth regulation, forming a pleiotropic model of its early stages. Alignment with emerging literature confirmed many predictions derived from early-stage Alzheimer’s disease’ CDM.

**Conclusions:**

A flexible approach for high-throughput data analysis, the Compact Disease Model generation, allows extraction of meaningful, mechanism-centered gene sets compatible with instant translation of the results into testable hypotheses.

## Background

In developed economies, the costs of medical services are constantly rising, stifling the growth and projecting to become unsustainable if the trend remains unchanged [1,2]. Some solutions propose the shift of the focus to early diagnostics of the diseases with the highest societal impact, to designing the strategies for reliable risk assessment and to tailoring prophylaxis efforts to the highest risk groups [3]. Another approach seeks to streamline the process of drug development by focusing the effort on the most promising targets and pre-clinical drug candidates. Both solutions may be assisted by the methods of bio- and chemo-informatics that operate within the realm of systems biology [4-6].

The most common type of the data analyzed by bioinformaticians is a set of differentially expressed genes obtained by microarray or RNAseq. Typical candidate list derived from these kinds of studies contains hundreds to thousands differentially expressed genes. However valuable, these sets are riddled with false-positives that changed their expression levels due to compensation for an overall increase in cellular stress or as a secondary effect of certain regulatory events, for example, the suppression of transcription factor’ activity or the shift in histone modification landscape. In other words, the differential expression of given gene often is a passive consequence of stress rather than a critical event directly contributing to disease pathogenesis.

Obviously, the focus of the research efforts should be on genes most essential in pathogenesis of given disease. However, this focusing is not trivial, as every chronic disease is studied by multiple research groups that customarily formulate multiple competing hypotheses [7], thus, populating the lists of potential candidates with thousands of entries. For Alzheimer’s disease alone, the Gene Cards compiled by Weizmann Institute of Science list 1890 molecules of relevance [8]. With < 25,000 genes known to comprise a genome, and no more than a third of them being expressed in a single tissue [9], this number is indicative that the long gene lists of today reflect rather poor target prioritization. Thus, there is a need for highly prioritized shortlists of potential targets directly linked to major pathogenic processes. Such lists, contracted by ontological enrichment, re-expanded by interaction network and validated by network clustering and alignment with literature were termed here Compact Disease Models (CDMs).

The reproducibility of a result in an independent experiment with at least slightly varied technical settings is the typical verification criterion for any scientific derivation [10]. In accordance to that, the gene-specific probes differentially expressed in the same direction in independently analyzed multiple subsets of the same experimental dataset and also in different experiments are less likely to report noise. Filtering of biological signals by consistency of gene-expression changes already demonstrated its value for enrichment analyses of genes mechanistically important for tumorigenesis [11,12] and metastasis [13]. As compared to gene lists generated using t-test, the lists generated using consistency of differential expression are target-enriched [4,11-14] and, thus, are more mechanistically relevant. For example, a reliable cancer mortality signature was produced by meta-analysis for the consensus changes observed in a variety of experiments across a number of model organisms [15]. An enrichment of gene expression signatures with mechanistically relevant targets was also attempted for neurodegeneration studies, such as Alzheimer’s and Parkinson’s diseases [16].

While any enrichment technique is capable of demonstrating the target enrichment, the utility of this enrichment is determined by the Receiver Operating Characteristic (ROC) of the process and the point of ROC cut-off. Importantly, non-overlapping components of individual datasets may be disease-specific while remaining related to pathogenic mechanism. Therefore, the requirement of consistency has to be imposed with minimally stringent cut-offs [17]. Here we present an approach that provides an improvement over previously described techniques. In that, we implemented sorting out the gene lists into the Consistency Tiers, thus, gaining control of the extent of information loss in the non-overlapping sub-sets. Each Tier can be assessed further by functional enrichment and alignment with independent literature data. The optimal size of the consensus signature could be selected depending on the nature of the disease [17]. All together, our process includes a three-step noise filter comprised of 1) prioritization of candidates by consistency of reported directional gene expression changes, 2) functional enrichment and 3) co-clustering of candidates in a network [18].

The resultant Compact Disease Model (CDM) provides a significant saving of research effort. Assuming N independent platforms being included in given analysis and m intersections needed to provide a robust mechanism-related gene list, the number of potential contributions becomes:

(1) $$\mathrm{REC}={C_{N}}^{m}\;P\;\left(m\right)$$

where REC – is the recall number (the number of totally available true positives), C_N_ ^m^ is the number of contributing platform combinations, P(m) is the number of strong mechanistic associates extracted per a single platform combination. In this case, the multi-platform nature of analyzed datasets would compensate for a low ROC curve area observed due to low recall (yield) component, while enrichment is high. Additional platforms to consider are comparative proteomics and quantitative PCR studies, massive parallel genome sequencing, promoter methylation arrays or others. To further improve the recall rate and polish the mechanistic details, the aggregated gene lists produced by all platforms combined are subjected to an interaction network building algorithm, sub-network identification and detailed assessment of the most relevant sub-networks by literature review.

Compacting mechanistically relevant genes into distilled shortlists may have a major impact on the routine verification of individual mechanistic hypotheses. Assume that a hypothesis H assigns correct connectivity between the functions X, Y, Z, the X being a receptor, Y being a G-protein and Z being a kinase. Relevance of the gene list is measured by factor of q, where q is the decimal fraction of bona-fide mechanistically relevant genes in the total list. The assignment will receive experimental verification only if all members X, Y, Z are bona-fide mechanistically related. For a 3-member sequence, the relationship is PEC = q^3^ which can be generalized into:

(2) $$\mathrm{PEC}=q^{n}$$

where PEC (probability of experimental correctness) is the probability that the mechanistic hypothesis is correct for a n-member sequence; q is the distillation factor of the list, n is the number of steps in a sequential mechanistic hypothesis. Under all other factors and techniques being equal, the exclusion of false positives from the gene lists is especially important for the mechanisms studied to a lesser degree (low g) and for complex hypotheses (high n).

To test CDM approach, we selected an example of Alzheimer disease, the most common form of adult-onset dementia. We were especially interested in addressing the earliest stages of this disease, when the pathological changes are still reversible and/or preventable. The particular focus of our analysis was at previously demonstrated anti-Alzheimer effects of anti-hypertensive drugs [19-22]. In our study, an application of CDM resulted in the distilled, tiered list of Alzheimer’s disease-related genes integrated into a biological network model. As potential players in early disease, a group of genes that encode proteins associated with traffic vesicles, oncogenes, G-protein regulators, gonadotropin hormones and insulin-related signaling molecules was identified. Insights gained by an analysis of this CDM may aid in shifting the therapeutic efforts to the reversible stages of neurodegenerative disease, when the neuronal damage is mild and self-perpetuating misfolded protein oligomers do not yet form.

## Methods

### Selection of datasets

Datasets for the study included A) GSE5281 on GPL570 HG-U133 Plus 2 Affymetrix Human Genome U133 Plus 2.0 Array including 71 normal controls and 91 disease related samples (N = 162); B) GSE15222 on GPL2700 Illumina Sentrix Human Ref-8 Expression Bead Chip, including 187 normal controls and 176 disease samples (N = 363); and C) GSE26927 on GPL6255 Illumina human Ref-8 v2.0 expression bead chip platform, including 58 normal controls and 60 disease samples (N = 118). The latter dataset comprises differential expression data covering several neuropathies: Alzheimer’s disease; Amyotrophic lateral sclerosis (ALS); Huntington Disease (HD); Multiple Sclerosis (MS); Parkinson Disease (PD); and Schizophrenia (SHIZ) of approximately equal size. The patient histories and disease severities were extracted from the information that accompanies the public domain datasets at GEO, NCBI at http://www.ncbi.nlm.nih.gov/geo/. Other datasets covering Alzheimer’s disease and dementia on GPL96 and GPL90 Affymetrix platforms were explored but not included due to failure to pass the quality controls, namely, large number of missing genes, evidence of data imputation or evidence of weak hybridization/weak signal. The primary data describing datasets A, B and C are presented in Additional file 1.

### Forming of a distilled gene list (CDM): consistency profiling step

The dataset GSE5281 comprises several distinct tissue subsets: EC - entorhinal cortex; HIP - hippocampus; MTG - Medial Temporal Gyrus; PC - Posterior Singulate; SFG - Superior Frontal Gyrus; VCX - Primary Visual Cortex, each being comprised of control and Alzheimer’s disease samples. The expression values were averaged for each anatomical locus for norm and disease. The averaged signal intensities were sorted by their magnitude and the upper 40% of the entries were included in the analysis on assumption that the expression levels for the remaining low-intensity signals is unlikely to exceed experimental noise. The ratios of the averages produce either up-regulated or down-regulated fold change values. The primary data were subjected to 2-tail, different variance hypothesis T-tests between normal control and disease subsets for each brain tissue type. The p-values of these T tests were converted into negative logarithms and the logarithms were averaged across all *tissue types*. For GSE5281, these averages formed the Primary Consistency Scores (PCSs). In addition to differential expression, for each gene, absolute expression levels were also tracked.

The dataset GSE15222 comprises normal controls separated into two subsets, numbers 1–85 and 86–178. The disease samples were also separated into two subsets, numbers 1–85 and 86–176. Absolute averaged expression levels were computed for each normal and disease subset, separately. Similarly to analysis of previous dataset, the upper 40% of entries by their expression level intensities were considered significant and included in the analysis. The difference in expression between the normal and disease subsets was assessed by T-tests as described above to compare each disease subset to each normal subset, four separate values were produced, and the negative decimal logarithms of T-test p-values were computed. The average of 4 negative logarithms formed Primary Consistency scores for GSE15222.

All gene-specific labels in GSE5281 and GSE15222 were ranked according to their Primary Consistency scores (PCSs) and the top 10% were selected. The highest ranking probes in GSE5281 and GSE15222 were assigned to a Consistency Tier 3, if the functionally related molecules (members of the same pathway) were also displaying high PCS. Assignment to Consistency Tier 2 was made in either of two situations: (a) two Affymetrix probes representing the same gene were displaying high rank PCS, and the direction of differential change was the same for both probes (all down-regulated or all up-regulated) in the group; (b) Affymetrix and Illumina probes representing the same gene were displaying high rank PCS and the direction of differential change was identical for both platforms. Consistency Tier 1 was assigned if either of three situations: (a) to the genes that displayed high PCS on both Affymetrix and Illumina platforms as well as multiple probes on Affymetrix platform, when the direction of differential expression changes was the same for all gene-specific probes; (b) to the probes that simultaneously qualify for Tier 3 and Tier 2; (c) to the three or more probes on Affymetrix platform that simultaneously showed high PCS ranking and the direction of expression changes was identical for all probes. Tiered consistency scores for all scored genes are available as the dataset D of the Additional file 1. The Tier 0 was produced by overlapping the Tier 1 and Tier 2 genes with the highest PCS ranks of GSE26927, thus, identifying a group of genes commonly participating in a number of neuropathies in addition to Alzheimer’s disease.

### Forming of a distilled gene list (CDM): ontological enrichment analysis step

Quantitative ontological analysis was performed using GO-MINER tool (http://discover.nci.nih.gov/gominer/index.jsp) using high-throughput online computing option at http://discover.nci.nih.gov/gominer/htgm.jsp. This technique measures preferential enrichment of the differentially expressed gene lists in one or more of approx. 9300 functional categories, organized in a tiled partially overlapping manner, with smaller specific categories merging into greater ones. To compute enrichment in a given category, the genes that are known to be related are tracked in the differential expressed gene list and in the total list. The enrichment coefficient can be estimated as:

(3) $$\mathrm{ENR}=\left[\mathrm{CG}/L\right]/\left[\mathrm{TG}/T\right]$$

Where: ENR – enrichment coefficient, CG – the number of genes with detected expression changes in a given functional category in the experimental gene list L, L – the number of genes in the experimental gene list, TG – total number of genes in a given functional category, T – the total number of genes assessed. Robustness of the enrichment coefficients is established by permuting the composition of L and expressed as p-value and False Discovery Rate (FDR). The current implementation of GoMiner uses a one-sided p-value calculated from a Fisher’s exact test. To get a low p-value, good enrichment and a fairly large size of category are required. The FDR approach addresses the multiple comparison problem, and protects against over-interpreting p-values that do not have a biological meaning.

The combination of Affymetrix and Illumina probe populations was used as the “Total file” or T. Since highly expressed genes are more likely to produce consistent differential expression signatures, the total file (T) was normalized to ensure equal average expression level as compared to the gene lists (L) under study, compensating this bias. Specifically, the total list in each case was ranked by expression levels and the upper rank populations of T producing equal averages to the given L were retained as expression-adjusted total files, and the rest were discarded from the analysis, effectively decreasing the number of genes in T. Tier 0, Tier 1, Tier 1 + Tier 2 and Tier 1 + Tier 2 + Tier 3 gene lists were used as “Changed file”. An option “All” was elected for “Data source”. Evidence Code was elected as “All”, accepting either experimental, curator inferred or computed data of functional involvement. Lookup setting for gene searching in the GO Consortium database was accepted as achieved by both cross-referencing and by use of synonyms. Both p-value of a functional enrichment category and false discovery rate (FDR) were elected as statistical criteria for including the qualifying genes in the summary report. The prospective functional enrichment categories were validated by 100 randomization cycles according to GO-MINER protocol. The smallest size for a functional enrichment category was accepted as 5. The GO-MINER output was sorted by FDR with the cutoff FDR < 0.2. The functional categories with the lowest false discovery rates were re-sorted by enrichment coefficients in the descending order. The relative functional enrichment coefficients reflect the extent of association of the differentially expressed genes with the pathological mechanism that caused the differential expression event in first place. The outputs of GO-MINER ontological analysis to the genes within Consistency Tiers 0–3 are available in the Additional files 2, 3, 4 and 5 datasets G-J.

The extent of ontological enrichment provides a cut-off for selection of the Consistency Tier levels to be submitted to network association step. The Tier 1 provided a conditionally optimal ROC cut-off due to high ontological enrichment and preserved pathway diversity. The Tier 1 + 2 + 3 was accepted, but considered less preferential due to a substantially lower proportion of mechanistically relevant genes based on ontological enrichment step.

### Forming of a distilled gene list (CDM): network analysis step

To organize sets of genes into biological networks, Ingenuity Pathway Assistant (IPA) tool was utilized (http://ingenuity.com/). Briefly, the tool places a gene list in the context of experimental and computed interactions systematized in its database. The functional links between the members of a gene list under study form a network with high clustering coefficients for members of the same biological pathway while clustering coefficients for random associates are low. Indeed, the members of the same pathway must be functionally associated with multiple other members of the same pathway, directly or via intermediates, thus producing non-random clustering. The extent of observed clustering is compared with a random model and the extent of observed clustering is expressed as a p-value of a network. The p-value matches a probability that the associations in the network have emerged randomly. The network is partitioned into sub-networks based on global optimization of clustering when a gene under consideration is shifted between the sub-networks as a test. The formed sub-networks are ranked based on the score, the latter being the negative decimal logarithm of sub-network non-randomness p-values.

The sets of sub-networks were built using gene lists comprising Consistency Tier 1 and a joint list comprised of all three numbered Tiers (Tier 1+ Tier 2 + Tier 3), the latter as a benchmark control to illustrate the loss of the priority rank by the sub-network comprising the genes relevant to neurological diseases. In each analysis, the highest ranking sub-networks were selected, merged and plotted as connectivity graphs. The genes displaying experimentally observed differential expression were co-plotted with sets of known network interaction partners. The possible network hubs were expanded, producing additional connections to more distant members. The Additional files 6 and 7 comprise the sub-network compositions for the Tier 1 and (Tier 1 + Tier 2 + Tier 3), including both experimental and inferred members.

### Validation of CDM by semantic tag enrichment analysis

The quantitative evaluation of enrichment of the microarray-derived dataset with literature-derived associations was applied as a validation criterion. Each gene lists was converted into Boolean [OR] statement, for example: [gene name 1] OR [gene name 2] OR …etc. and used as a search query in Pubmed. The hits produced by PubMed were defined as Total. Additional delimiting search queries were imposed: A. [(disease or pathology or disorder)]; B. [cancer]; C [(disease or pathology or disorder) and stress; D. [(disease or pathology or disorder) and (Alzheimer’s or Alzheimer or neuropathy or neuropathic or neuro-degeneration or neurodegeneration or neurodegenerative or dementia)]. The numbers of hits for each delimited strategy were enumerated and related to the number for the total list based on gene names only. Variation in the datasets was taken into account by dividing each consistency tier list into subsets and repeating the procedure independently for each subset, pooling the variation and distributing it equally per each subset (within each consistency tier).

In this technique, both [cancer] and [(disease or pathology or disorder)] strings were used as controls accounting for non-specific organism or tissue-level stress that generally accompanies any severe pathology, while the string [(disease or pathology or disorder) and stress] was controlling for explicit gene association with stress in pathological conditions and the string [(disease or pathology or disorder) and ([Alzheimer’s] or (Alzheimer or neuropathy or neuropathic or neuro-degeneration or neurodegeneration or neurodegenerative or dementia)] was controlling the expected specific association of the gene lists and the disease of interest.

## Results

### Overview of differential expression consistency in Alzheimer’s disease

Affymetrix GPL570 platform comprises approximately 54000 probes, while Illumina platform comprises ~22000 probesets. Of the ~24000 independent expressed genes measured by both platforms, 78 sets were satisfying criteria of the Tier 0, 105 sets were satisfying the criteria of the Tier 1, 85 sets were satisfying the criteria of the Tier 2, 450 sets matched the Tier 3 and 1298 sets were demonstrating high PCS without being validated by other consistency criteria. On both platforms, the genes within the top 40% range by their absolute expression served as random control. Of the 190 probe-sets in Tier 1+ Tier 2, the Tier 0 comprised 78, indicating that > 40% of genes robustly reported as being differentially expressed in Alzheimer’s disease also produced robust detection in other neuropathies in agreement with [16]. In all Consistency Tiers, the fold differences of differential expression effects were relatively small, rarely exceeding 3-fold. In Tier 1, 20 out of 105 probe-sets were up-regulated and the remaining 85 being down-regulated. In Tier 2, 12 out of 97 probe-sets were up-regulated, the remaining 85 being down-regulated. In Tier 3, the down-regulated pattern was shown by 316 genes and 176 genes were up-regulated. In the high PCS/unconfirmed group, 715 genes were down-regulated and 570 were up-regulated. In random control, the ratio of up and down-regulation signals was close to 1. The extent of relative down-regulation was strongly correlating with the extent of differential expression detection consistency. These numbers show a greater tendency for down-regulation in Alzheimer’s disease-related genes and support functional significance of the consistency profiled gene lists, in agreement with degenerative character of the Alzheimer’s process [23].

The absolute expression levels were also positively correlating with consistency of differential expression detection. Thus, Tier 0 average signal was ~4300 arbitrary units, the remaining (Tier 1+ Tier 2) signals were, after exclusion of Tier 0, at ~2330 arbitrary units, while the random control genes were at ~1725 arbitrary units for the top 40% of ranked intensities and ~730 arbitrary units for the entire array (see the Dataset C in Additional file 1). The 3 to 5-fold increase in average absolute expression in the Consistent gene lists vs. Random Control may be an artifact: the genes with higher expression levels may also display higher signal-to-noise ratio at hybridization. Also, at a higher concentration of transcript the thermodynamic quotient and Gibbs energy of binding increases. For genes with higher expression levels the relative proportion of binding at non-specific sites is lower. However, it is also known that mechanistically important targets tend to be the hubs of the biological network that also tend to be expressed at higher levels than non-hub entities [24,25]; this feature of biological networks provides additional robustness [26,27]. However, to account for possible gene intensity bias, further functional enrichment analysis was conducted after respective normalization.

**Figure 1:**
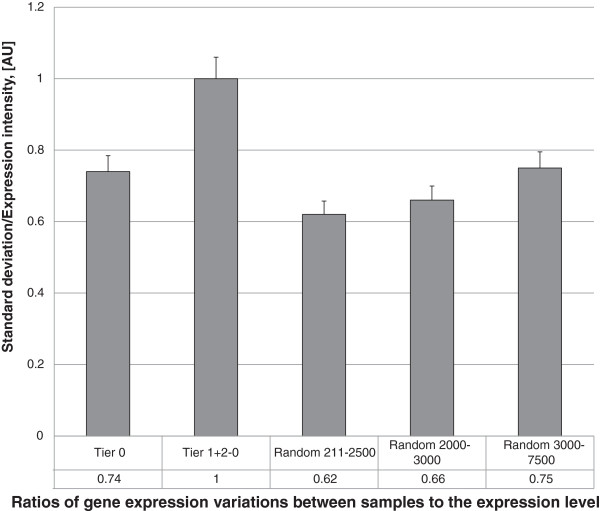
**A. Tier 0 and Tier (1 + 2) genes differentially expressed in Alzheimer’s disease and other neuropathies are compared with significantly expressed random genes on Illumina platform, dataset C.** Tier 0 is produced by an overlap of Tier (1 + 2) in Alzheimer’s disease panel (Datasets A and B) with the multiple neurodegeneration disease panel (Dataset C). Tier (1 + 2–0) is produced by the balance of Tier (1 + 2) genes with the subtraction of Tier 0. Random genes ranked by intensity were sampled based on the position in the rank. Expression intensities in the groups of genes formed as described above were measured and plotted.

Another concern was a possibility of a bias due to decreased inherent variation within consistently reporting gene sets, as could be expected for tightly regulated pathways. If this is true, the consistency in differential expression of these genes would reflect not a prevalence of their biological relevancy, but lower levels of respective backgrounds. To rule this scenario out, variations were measured among all Consistency Tiers and were compared to variations observed in Random Control genes both globally and in the subsets selected by matching of their expression intensities. The results are presented in Figure 1.

This analysis points at *higher* variation in consistent differentially expressing datasets. Thus, the biased scenario was ruled out and the consistency of the gene expression changes, indeed, was found to reflect the difference between the disease and the norm.

**Figure 2:**
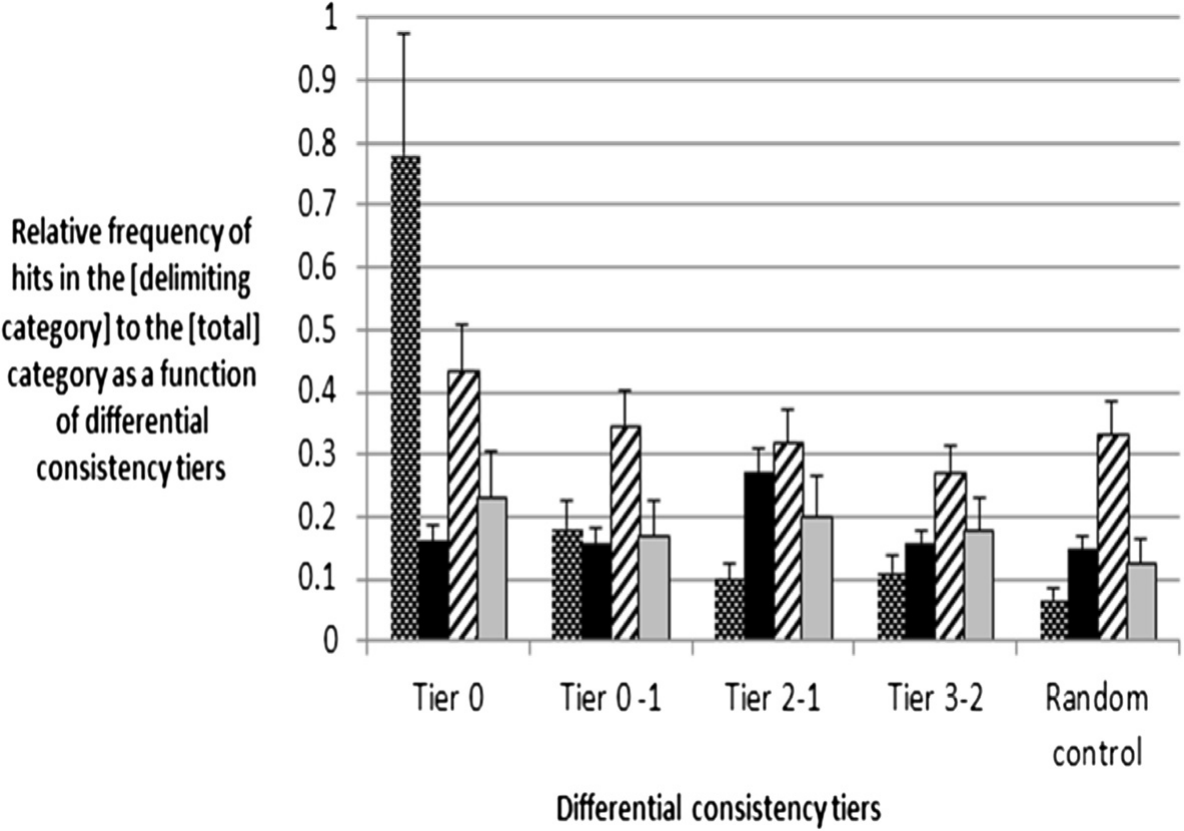
**Dependence of the relative enrichment in literature-inferred gene roles as a function of detection consistency.** For each gene list (Tier 0, Tier 0–1, Tier 2–1, Tier 3–2, Random control), the gene symbols were converted into a Boolean representation (simply connected by the operator [OR]). Each Boolean-converted list was used as a query in Pubmed and the number of hits was detected. The primary query for each gene list was modified by 4 sub-queries, from left to right: Checkered bars: [gene list] + [(disease or pathology or disorder) and (Alzheimer’s or Alzheimer or neuropathy or neurodegeneration)]; black bars: [gene list] + [cancer]; striped bars: [gene list] + [(disease or disorder or pathology)]; grey bars: [gene list] + [(disease or pathology or disorder) and stress]. The modified queries produced the numbers of hits smaller than the number of hits produced by un-delimited gene list in Boolean form. The ratios of the database responses for the modified vs. unmodified query were plotted for each group of four bars representing a consistency tier. The relative frequencies (ratios) for the queries [(disease or pathology or disorder) and stress] and [(disease or pathology or disorder) and (Alzheimer’s or Alzheimer or neuropathy or neurodegeneration)] were multiplied by 10 for convenience of representation and analysis. The Tiers 0–3 represent the lists of genes obtained as disclosed in the Methods; the Tier 1–0 is the result of subtracting the Tier 0 list from the Tier 1 list; the Tier 2–1 is the result of subtracting the Tier 1 from the Tier 2 list; the Tier 3–2 is the result of subtracting the Tier 2 from the Tier 3 list; the Random control set was obtained by randomly selecting the genes among Affymetrix and Illumina total lists and the list is not expression intensity normalized.

Still, there might be a concern that the differentially expressed data represent stress responses at both organism and tissue-specific levels, in other words, the responses expected to be pertinent to any severe pathology rather than to reflect a disease-specific mechanism. Figure 2 shows the extracted Consistency Tiers as analyzed by the methodology described above. The method comprises the Boolean presentation of the gene list crossed with the delimiting statement reflecting either association with a non-specific stress or with a specific disease. The statements like [(disease or disorder or pathology)] crossed with the corresponding Boolean representations of the consistency tiers would locate the literature publications associating the genes of interest with any disease, non-specific to the study. The query statements like [cancer] crossed with the corresponding Boolean representations of the consistency tiers would locate the literature publications associating the genes of interest with cancer as another proxy for a non-specific multiple roles played by many signaling molecules. The statement relating the gene list of interest to stress-response comprises the negative control. Thus, relative enrichment of the disease-specific vs. disease non-specific PubMed hits for certain levels of consistency would represent a measure of ensuring the mechanistic involvement of the genes in the disease-specific pathogenesis Figure 2 represent Venn-transformed enrichment diagrams for all Consistency Tiers.

Based on the results presented in Figure 2, it is apparent that only the Tier 0 produces a highly enriched disease associated gene list, the Venn Tier 1–0 is still significantly more enriched than the Random Control gene list, while an enrichments in Venn Tiers 2–1 and 3–2 were marginal. In fact, the enrichment for the delimiter query [(disease or pathology or disorder) and (Alzheimer’s or Alzheimer or neuropathy or neurodegeneration)] in the Tier 0 was approximately 10 fold as compared to the Random Control. The degrees of enrichment against all non-specific disease-related controls were similar in each Consistency Tier and remained within a margin of experiment error. All together, against the non-delimited gene list’ background and against the panel of negative controls, the disease-specific genes demonstrate the relative enrichment of ~ 10 in the Tier 0, ~3 in the Tier 1–0, ~1.5-2 in the Tiers 1–2 and 3–2.

In its composition, the Tier 3 roughly corresponds to the target enrichment achievable in a benchmark (traditional) gene expression analysis based on T-test alone (Figure 2). Low literature tag enrichment in consistency Tier 3 and in all tiers below Tier 3 questions the meaningfulness of the data not pre-cleaned by triple filtration of CDM or similar knowledge-based techniques. The data of Figure 2 show that the information most useful for mechanism-inferring purposes contained within Tier 1 and, in some case, Tier 0.

### Functional enrichment analysis

Specific pathological mechanisms manifest by differential expression of genes that belong to just a few selected pathways. The effected functional categories may develop high and statistically significant enrichment coefficients in the changed gene list. The higher the extent of enrichment, the stronger is the link between the disease mechanism and the functional category of interest, pointing to greater specificity of the signal. Table 1 below combines expression-normalized functional enrichment coefficients computed for Top 20 most enriched ontological categories for gene lists in the Tiers 0–3.

**Table 1:** Enrichment coefficients for top significance functional categories in different Consistency Tiers

**Tier**	**Go category**	**TG**	**CG**	**ENR**	**LOG10 (p)**	**FDR**
0	GO:0019894_kinesin_binding	8	4	15	−4.1	0.011
0	GO:0007269_neurotransmitter_secretion	38	7	5.7	−3.8	0.018
0	GO:0030426_growth_cone	30	6	6	−3.5	0.024
0	GO:0008021_synaptic_vesicle	42	7	5	−3.5	0.030
0	GO:0007204_elevation_of_cytosolic_calcium_ion_concentration	20	5	8	−3.5	0.028
0	GO:0051480_cytosolic_calcium_ion_homeostasis	22	5	7	−3.3	0.03
0	GO:0051279_regulation_of_release_of_sequestered_calcium_ion_into_cytosol	6	3	15	−3.2	0.05
0	GO:0030672_synaptic_vesicle_membrane	24	5	6.4	−3.1	0.06
0	GO:0010522_regulation_of_calcium_ion_transport_into_cytosol	7	3	13.	−3.0	0.06
0	GO:0048854_brain_morphogenesis	7	3	13	−3.0	0.0
0	GO:0050852_T_cell_receptor_signaling_pathway	15	4	8	−3.0	0.06
0	GO:0051648_vesicle_localization	16	4	8	−2.9	0.06
0	GO:0051209_release_of_sequestered_calcium_ion_into_cytosol	8	3	12	−2.8	0.06
0	GO:0051282_regulation_of_sequestering_of_calcium_ion	8	3	12	−2.8	0.06
0	GO:0051283_negative_regulation_of_sequestering_of_calcium_ion	8	3	12	−2.8	0.06
0	GO:0002429_immune_response-activating_cell_surface_receptor_signaling_pathway	17	4	7	−2.7	0.06
0	GO:0002768_immune_response-regulating_cell_surface_receptor_signaling_pathway	17	4	7	−2.7	0.07
0	GO:0050851_antigen_receptor-mediated_signaling_pathway	17	4	7	−2.7	0.07
0	GO:0007281_germ_cell_development	29	5	5	−2.7	0.07
0	GO:0030594_neurotransmitter_receptor_activity	9	3	10	−2.6	0.08
1	GO:0060198_clathrin_sculpted_vesicle	5	3	19	−3.6	0.03
1	GO:0019894_kinesin_binding	9	5	18	−5.5	0.004
1	GO:0042288_MHC_class_I_protein_binding	6	3	16	−3.3	0.05
1	GO:0042287_MHC_protein_binding	7	3	14	−3.0	0.06
1	GO:0051279_regulation_of_release_of_sequestered_calcium_ion_into_cytosol	7	3	14	−3.0	0.06
1	GO:0005834_heterotrimeric_G-protein_complex	12	5	13	−4.8	0.0049
1	GO:0010524_positive_regulation_of_calcium_ion_transport_into_cytosol	5	2	13	−2.0	0.12
1	GO:0035267_NuA4_histone_acetyltransferase_complex	5	2	13	−2.0	0.12
1	GO:0043113_receptor_clustering	5	2	13	−2.0	0.12
1	GO:0045576_mast_cell_activation	5	2	13	−2.0	0.12
1	GO:0046173_polyol_biosynthetic_process	5	2	13	−2.0	0.12
1	GO:0051322_anaphase	5	2	13	−2.0	0.12
1	GO:0051668_localization_within_membrane	5	2	13	−2.0	0.12
1	GO:0010522_regulation_of_calcium_ion_transport_into_cytosol	8	3	12	−2.8	0.06
1	GO:0031177_phosphopantetheine_binding	8	3	12	−2.8	0.06
1	GO:0008277_regulation_of_G-protein_coupled_receptor_protein_signaling_pathway	12	4	11	−3.5	0.03
1	GO:0008298_intracellular_mRNA_localization	9	3	11	−2.7	0.07
1	GO:0032410_negative_regulation_of_transporter_activity	9	3	11	−2.7	0.07
1	GO:0010676_positive_regulation_of_cellular_carbohydrate_metabolic_process	6	2	11	−1.9	0.14
1	GO:0045298_tubulin_complex	6	2	11	−1.9	0.14
1+ 2	GO:0019894_kinesin_binding	9	6	12	−5.7	0.007
1+ 2	GO:0060198_clathrin_sculpted_vesicle	5	3	11	−2.8	0.06
1+ 2	GO:0008298_intracellular_mRNA_localization	9	5	10	−4.4	0.009
1+ 2	GO:0005871_kinesin_complex	8	4	9	−3.3	0.03
1+ 2	GO:0042288_MHC_class_I_protein_binding	6	3	9	−2.5	0.08
1+ 2	GO:0045298_tubulin_complex	6	3	9	−2.5	0.08
1+ 2	GO:0005834_heterotrimeric_G-protein_complex	12	5	7.5	−3.5	0.02
1+ 2	GO:0005881_cytoplasmic_microtubule	15	5	6	−3.0	0.04
1+ 2	GO:0008088_axon_cargo_transport	15	5	6	−3.0	0.04
1+ 2	GO:0008277_regulation_of_G-protein_coupled_receptor_protein_signaling_pathway	12	4	6	−2.5	0.08
1+ 2	GO:0014047_glutamate_secretion	12	4	6	−2.5	0.08
1+ 2	GO:0032182_small_conjugating_protein_binding	16	5	5.6	−2.9	0.045
1+ 2	GO:0043130_ubiquitin_binding	16	5	5.6	−2.9	0.045
1+ 2	GO:0006458_‘de_novo’_protein_folding	26	8	5.5	−4.3	0.009
1+ 2	GO:0006941_striated_muscle_contraction	13	4	5.5	−2.3	0.09
1+ 2	GO:0072384_organelle_transport_along_microtubule	13	4	5.5	−2.3	0.09
1+ 2	GO:0051084_‘de_novo’_posttranslational_protein_folding	24	7	5	−3.6	0.02
1+ 2	GO:0005876_spindle_microtubule	18	5	5	−2.6	0.07
1+ 2	GO:0005200_structural_constituent_of_cytoskeleton	33	9	5	−4.3	0.01
1+ 2	GO:0010970_microtubule-based_transport	26	7	5	−3.4	0.02
1+ 2+ 3	GO:0033180_proton-transporting_V-type_ATPase__V1_domain	7	6	6	−4.2	0.0018
1+ 2+ 3	GO:0004708_MAP_kinase_kinase_activity	6	5	6	−3.4	0.006
1+ 2+ 3	GO:0042288_MHC_class_I_protein_binding	6	5	6	−3.4	0.006
1+ 2+ 3	GO:0042777_plasma_membrane_ATP_synthesis_coupled_proton_transport	6	5	6	−3.4	0.006
1+ 2+ 3	GO:0030897_HOPS_complex	5	4	5	−2.7	0.025
1+ 2+ 3	GO:0031338_regulation_of_vesicle_fusion	5	4	5	−2.7	0.025
1+ 2+ 3	GO:0035542_regulation_of_SNARE_complex_assembly	5	4	5	−2.7	0.025
1+ 2+ 3	GO:0060198_clathrin_sculpted_vesicle	5	4	5	−2.7	0.025
1+ 2+ 3	GO:0046933_hydrogen_ion_transporting_ATP_synthase_activity__rotational_mechanism	15	11	5	−6.3	0
1+ 2+ 3	GO:0046961_proton-transporting_ATPase_activity__rotational_mechanism	18	13	5	−7.2	0
1+ 2+ 3	GO:0004712_protein_serine_threonine_tyrosine_kinase_activity	7	5	5	−2.9	0.014
1+ 2+ 3	GO:0042287_MHC_protein_binding	7	5	5	−2.9	0.014
1+ 2+ 3	GO:0033178_proton-transporting_two-sector_ATPase_complex__catalytic_domain	17	12	5	−6.5	0
1+ 2+ 3	GO:0019894_kinesin_binding	9	6	4.5	−3.2	0.008
1+ 2+ 3	GO:0010676_positive_regulation_of_cellular_carbohydrate_metabolic_process	6	4	4.5	−2.2	0.059
1+ 2+ 3	GO:0035493_SNARE_complex_assembly	6	4	4.5	−2.3	0.059
1+ 2+ 3	GO:0045261_proton-transporting_ATP_synthase_complex__catalytic_core_F(1)	6	4	4.5	−2.3	0.059
1+ 2+ 3	GO:0045739_positive_regulation_of_DNA_repair	6	4	4.5	−2.3	0.059
1+ 2+ 3	GO:0045913_positive_regulation_of_carbohydrate_metabolic_process	6	4	4.5	−2.3	0.059
1+ 2+ 3	GO:0009135_purine_nucleoside_diphosphate_metabolic_process	8	5	4	−2.6	0.032

TG – total genes in a given functional category within the total list T, CG – changed genes within the list L, ENR – enrichment coefficient, LOG10(p) – logarithm of p-value of one-sided Fisher’s test of significance of a given ENR at a given group size, FDR – false discovery rate. Venn Tier 1 + 2 is the combination of the Tier 1 and Tier 2, Venn Tier 1+ 2 + 3 is the combination of the Tiers 1, 2 and 3.

In general, the enrichment coefficients positively correlate with consistency scores, decreasing in the direction from Tier 1 (highest consistency) to Tier 3 (lowest consistency). The trend reversal from Tier 0 to Tier 1 can be explained by significant reduction (by 90%) of the total gene number in the Tier 0 as a result of expression normalization. In the Top 20 categories, the enrichment coefficients were in the range of 4.5-19, with a tendency to an upper side of the range. In non-normalized datasets, the stringent normalization by absolute expression masks the extent of functional enrichment as it may reach the values of ~100 for Tier 0 and ~40 for Tier 1, thus, being far above the typical values observed in traditional microarray experiments [28]. Thus, much higher distillation coefficient q of the model (1)-(2) may be reached. Based on comparison of the functional enrichments in CDM approach and the benchmark exemplified by [28], the increase in network-assisted capability to infer relevant mechanisms may be quite dramatic and certainly merits further study.

Based on the Table 1, the function of traffic vesicle formation dominates in the Tiers 0 and 1 and Venn Tier 1 + 2. This function includes sub-functions of kinesin binding (synuclein-α; actin β; actin γ1; kinesin-associated protein 3), clathrine vesicle formation (synaptotagmin 1; synaptotagmin 13; synaptic vesicle glycoprotein 2B), calcium release (calmodulin 2; synuclein-α; thymus cell antigen 1, θ; cholecystokinin B receptor; guanine nucleotide binding protein (G protein), γ 3). Synaptic vesicle development categories were prominent in the Tier 0, while an axon development category was prominent in the Venn Tier 1 + 2 (synaptotagmin 1; synaptotagmin 13; synaptic vesicle glycoprotein 2B). Vesicle formation–related functionalities displayed the highest enrichment coefficients among all consistency tiers and were accompanied by the lowest p-values and FDRs. Microtubule and cytoskeleton development were prominent in the Tier 1 and Venn Tiers 1 + 2 and 1 + 2 + 3 (tubulin, γ complex associated protein 3; tubulin, γ complex associated protein 2; tubulin, β 3 class III; tubulin,β 2C; tubulin β, class I; tubulin, α1c; tubulin, α1b). A related function of cell motility was predominately populated by the molecules that relate to mast cell activation (tyrosine 3-monooxygenase/tryptophan 5-monooxygenase activation protein, ζ polypeptide; thymus cell antigen 1, θ; synuclein-α). Another prominent functional category, common to the Tiers 0–3, was MHC binding, receptor binding, ubiquitin targeting and other forms of protein binding mediated by proteasome subunits, cytoskeleton and chaperones (ubiquitin-conjugating enzyme E2N; p21 protein (Cdc42/Rac)-activated kinase 1; thymus cell antigen 1, θ; actin β; actin γ1). Regulation of G-protein signaling was highly enriched category in the most conserved Venn Tiers 0–2 (regulator of G-protein signaling 4; regulator of G-protein signaling 6; regulator of G-protein signaling 7; synuclein-α, calmodulin 2; cholecystokinin B receptor; γ-aminobutyric acid (GABA) B receptor, 2; guanine nucleotide binding protein (G protein), γ3). Less surprisingly, an importance of GABA neurotransmission was detected (γ-aminobutyric acid (GABA) B receptor, 2; γ-aminobutyric acid (GABA) A receptor, gamma 2), as well as related glutamate secretion (glutaminase; synaptotagmin 1; synuclein-α), neurotransmitter binding (cholinergic receptor, muscarinic 1; cholecystokinin B receptor; γ-aminobutyric acid (GABA) A receptor, γ2) and brain morphogenesis (platelet-activating factor acetylhydrolase 1b, regulatory subunit 1; McKusick-Kaufman syndrome; presenilin 2) functionalities. Remarkably, consistent datasets lacked amyloid β (A4) precursor protein APP. One possible explanation is that differential expression of APP monomer is negligible, while its pathological role unfolds at the level of toxic oligomers [29].

### Biological network modeling

**Figure 3:**
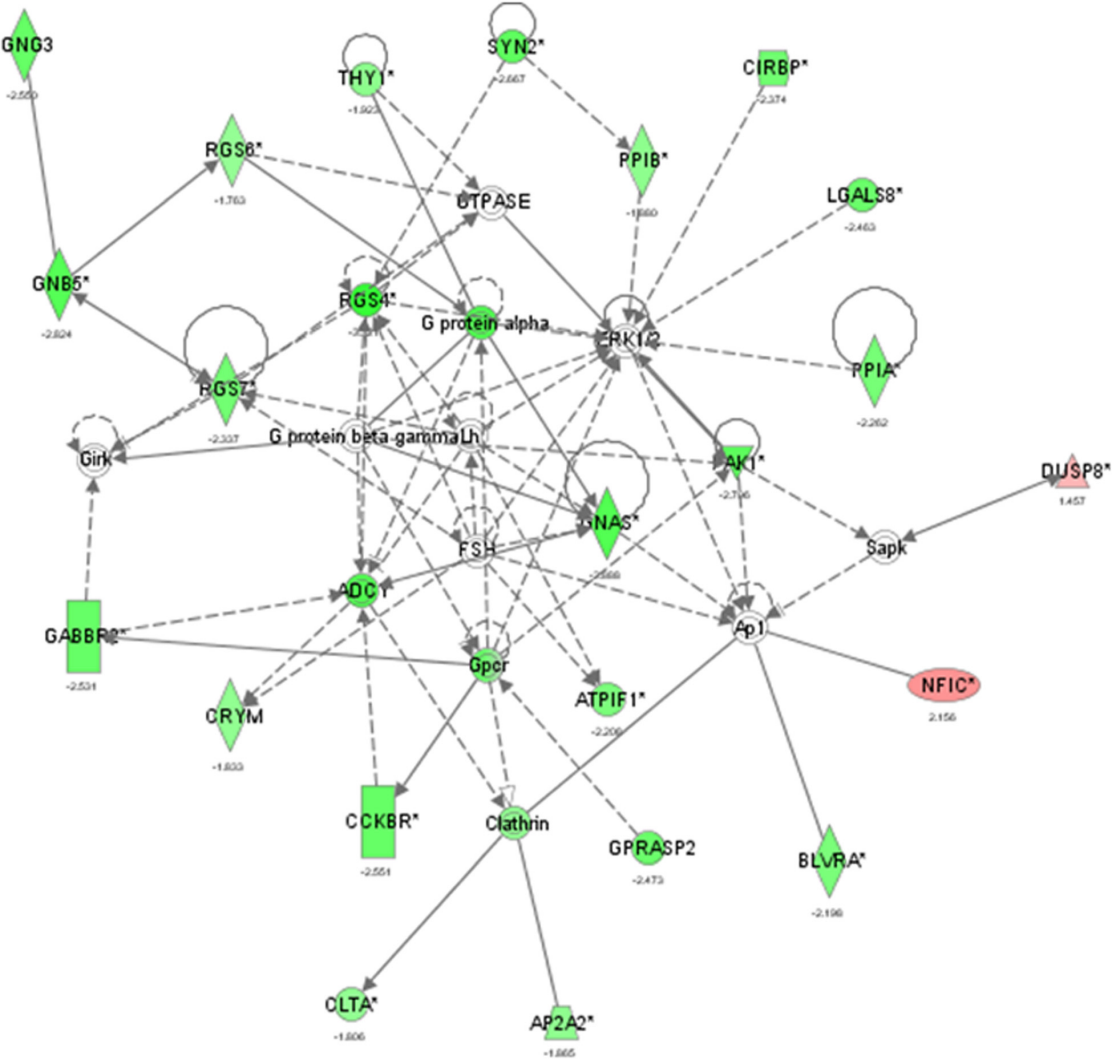
**Biological network-based model of interactions between the most essential Alzheimer’s disease-related genes representing the sub-network 1 of Table **2**.** Green figures indicate down-regulation, red figures indicate upregulation, grey figures mean unchanged expression levels. Rectangular figures indicate receptors, rombi –peptidases, triangles – kinases/phosphatases, circles –other; solid connecting line – binding only, solid connecting arrow – acts upon, dotted lines indicate indirect functional relationships (such as co-regulation of expression of both genes in cell lines).

The molecules populating Consistency Tier 1 that was optimal in terms of balance between functional enrichment and recall rate of the most relevant mechanistic participant entries were analyzed using Ingenuity Pathway Assistant (IPA) tool. The first order interactions were imputed automatically, the partners being the hubs of the cell signaling pathways in network proximity to the changed genes. The addition provided by the IPA network is valuable, since this feature partially compensates for low recall rate observed when the consistency criteria are applied. The distinctions between the sub-networks are algorithm-generated and therefore somewhat artificial. We retained for further analysis all significant hubs regardless of the sub-network they were assigned to by IPA. A sub-network 1 of the interaction network is shown in Figure 3 and the composition of the entire network is provided in Table 2.

**Table 2:** Composition and scoring of Alzheimer’s disease molecular sub-networks generated by IPA

**ID**	**Molecules in network**	**Score**	**Focus molecules**	**Top functions**
1	ADCY,Ap1,AP2A2,ATPIF1,BLVRA,CCKBR,CIRBP,Clathrin,CLTA,CRYM,DUSP8,ERK1/2,FSH, GABBR2,Girk,GNAS,GNB5,GNG3,GPRASP2, GTPASE,LGALS8,Lh,PAK1,PPIA,PPIB,RGS4,RGS6,RGS7,Sapk,SYN2,THY1,TNPO1	49	23	Neurological Disease, Reproductive System Development and Function, Cell Death
2	14-3-3,ACTB,ACTG1,Actin,ACTR1A,aldo,Alpha tubulin,ATP5C1,ATP5G1,ATP6V1D (includes EG:299159),ATP6V1E1,ATP6V1E2,Beta Tubulin,Cofilin,Dynein,EIF3K,ELAVL4,F Actin, GAPDH, H + −transporting two-sector ATPase, Hsp90,NFkB(complex),PFDN5,SMARCC1,SNCA,SORBS1,STMN2,TUBA1B,TUBA1C,TUBB3,TUBB,TUBB2C,Tubulin,Vacuolar H + ATPase,ZBTB20	47	22	Cellular Assembly and Organization, Cellular Function and Maintenance, Immunological Disease
3	26sProteasome,Akt,ATP8A2,BAG6,CD3,COPS4, COX5B (includes EG:100002384),DNAJB12, DNAJC6,DNAJC8,ERK,GABRG2,Hsp70,HSP,HSPA8,HSPB3,Ikb,Insulin,Jnk,Laminin,LSM14B,Mapk,NDFIP2,P38MAPK,Pka,PPME1,PSMD4, PTP4A2,SNRPN,Sos,SYT1 (includes EG:20979), TCR,UBE2N,Ubiquitin, YWHAZ	31	16	Cellular Compromise, Cell-To-Cell Signaling and Interaction, Cellular Growth and Proliferation
4	ADORA2A,ATP,ATP6V1H,CACNA1E,CACNA2D3,CACNB2,DYNC1I1,EIF1,EIF5B,FOS,GPR1 (includes EG:100004124),IDS,KDM5B,KIFAP3, KLF15,L-glutamic acid,LANCL1,MAP2K7,MAPK3, MYC,MZT1,PNO1,PPP1R7,RAD51C,REEP1,RPL15,SLC17A7,SLC17A,SRSF2,TGFB1 (includes EG:21803),TIMM23,TMEM97,TUBGCP2,TUBGCP3,XRCC3	29	17	Hematological System Development and Function, Hematopoiesis, Tissue Development
5	AGRP,APC,ARHGEF9,CHCHD3,CHCHD6,CYB5D1,EIF3C/EIF3CL,IDH3G,LAMA3,MC4R,MRPS7,MRPS22,NAV1,NAV2,NFIA,POLH,PRDM5,PSMA1,PSMB2,PSMB3,PSMB6,RPL5,RPL6,RPL17,RPL19,RPL30,RPL31,RPL10A,RPLP0,RPLP2,RUNX2,SLC35E1,UBC,UBL7,USP3	21	12	Connective Tissue Development and Function, Tissue Morphology, Genetic Disorder
6	ACACB,ATP5A1,ATP5C1,ATP5D,BBX,BCL2L1,CDC16,CUEDC1,CUL3 (includes EG:26554), DHX30,E2F4,FAM162A,GSK3A,HINT1,IL4 (includes EG:16189),KCNAB2,LAMP2,mir-451,NACC1,NFIC,OTUD7B,PEBP1,PRKCQ,PSEN1,RELA,RTN1 (includes EG:104001),SLC11A2, SMAD3,SYT13,TMEM85,TNFSF10,TPT1 (includes EG:100043703),TTK,TUBA3C/TUBA3D, ZNF83	21	12	Nucleic Acid Metabolism, Small Molecule Biochemistry, Cellular Compromise
7	FAM63A,NAA38	2	1	
8	GNB2L1,SLC9A6	2	1	Cell Cycle, Connective Tissue Development and Function, Developmental isorder
9	DDX19B,GADD45G,RWDD2B	2	1	Tissue Development, Cell Cycle, DNA Replication, Recombination, and Repair

Molecules in network comprise both experimentally discovered molecules and known/predicted close interaction partners. The Score is a measure of clustering coefficient between the sub-network components. Focus Molecules are the differentially expressed gene products with experimental evidence of the linkage to disease pathogenesis and are denoted by capital letters, while small letters designate inferred interactions.

According to the Table 2, the sub-networks 1 and 2 demonstrate significant scores associated with p-value of 10^-49^ and 10^-47^, respectively, where the score being the probability that the genes associated at this extent of clustering were drawn randomly. Per IPA functional assignment, the highest score sub-network 1 corresponds to neurological diseases and comprises vesicle-forming components in agreement with GO-MINER analysis, validating the CDM approach from the point of internal consistency. As a control, a gene list sampling equal to Tier 1 in size and selected from the T-test enriched population (benchmark method, no CDM processing) was analyzed. The control list prioritized different sub-networks and emphasized the pro-inflammatory pathways, but not the neurological disease-specific sub-network as does CDM list (data not shown). The same trend persisted across the levels of detection consistency from T-test only and up to the Tier 3. The sub-network 2 comprises mostly cytoskeleton and mitochondrial components. The sub-network 3 displays a score of 31 and comprises other components such as chaperones, ubiquitin pathway members and proteasome subunits. Sub-networks 4–9 were significant but displayed lower scores.

The abundance of oncogenes in the associated hub subset was remarkable. To quantify the extent of association with oncogenes, the symbol T was defined as Pubmed response to the gene symbol, assumed to be proportional to the total number of biological interactions mediated by the gene and its products. High T numbers correspond to the hubs of biological network.

To put it in a larger genomic context, a random sample of 118 gene names was extracted and the T-values were determined, producing 2 hits with 1000 < T < 5000, 2 hits with 5000 < T < 10000 and 1 hit with T > 10000. Based on this sampling and the total number of genes ~ 20000, an estimate of ~ 500 hubs with T > 5000 was shown for the human interaction network. In the network sample associated with the Tier 1 consistently expressed gene list, 36 hubs of the comparable connectivity was present per 96 network associates. This is not a remarkable finding, considering that Ingenuity databank is likely biased in favor of hub enrichment. However, the finding that the ratio of oncogenes to tumor suppressors is skewed toward oncogenes is counterintuitive for a degenerative disease.

To assess the background ratio of oncogenes vs. suppressors, several databases were enquired. Search of OMIM (http://www.ncbi.nlm.nih.gov/omim) leads to 647 hits responding to the query [(“oncogene or oncogenes”)], while 882 hits responded to the queries [(“tumor suppressor” or “tumor suppressors”)]. These numbers correspond to ~7:5 ratio of tumor suppressors to oncogenes in the global network. Similar search with the databases “Genes” and “Proteins” at NCBI produced ~1:2 ratios. An analysis of the database GeneCards at http://www.genecards.org leads to the ratio ~1:1 for the same queries. With that, an average ratio of ~0.8:1 of tumor suppressor to oncogenes may be assumed as a random global control.

This ratio markedly differs from the ratio observed in our data. Tumor suppressor/oncogene ratio in the hubs associated with neuropathy network was 2:9 (APC and TGFB1 as tumor suppressors, ERK1/2, AKT, MYC, FOS, AP-1, BCL2L1, HSP70, HSP90, RELA being pro-growth and oncogenic, while the MAPK3, MAPK8 and MAPK14 were marked as having context dependent dual functions). Except APC, none of the hits in this T range was associated with “tumor suppressor” label, while AKT, MYC, FOS, AP-1, BCL2L1, RELA were denoted as “oncogenes”.

We further tested if this oncogene association is limited to our data or is more general. A PubMed query [(Alzheimer’s or Alzheimer or neuropathy or neuropathic or neuro-degeneration or neurodegeneration or neurodegenerative or dementia)] was further delimited by the keywords [(“oncogene” or “oncogenes”)] as well as [(“tumor suppressor” or “tumor suppressors”)]. The ratio of 3.4:1 was observed, while a control query [(disease or disorder)] produced 1.9:1 ratio. Similar queries [neuropathy or neurodegeneration] and [dementia or “cognitive decline”] produced the ratio 3.5:1 above the random ~2:1, consistent with our data.

The high-T sub-population of network associates was segregated from the initial changed gene list (Tiers 0, 1–0, 2–1 combined) and all sub-populations underwent a similar analysis as above. Specifically, the corresponding gene lists were converted into Boolean queries and were delimited with “oncogene” and “tumor suppressor” terms. The Alzheimer’s related genes were compared with a random gene sample. The random control and the initial (non-tiered) list of differentially expressed genes demonstrated comparable oncogene/tumor suppressor hit ratios of 2:1, while the population of extracted network associates produced the hit ratio of 5.7:1. For sense of perspective, the corresponding ratio for oncogene BCL2-centered network was 4.6:1 and for tumor-suppressor-centered p53-centered network was 1:2.2. Considering these ratios, the Alzheimer’s disease network associates were as a group more oncogenic than the associates of BCL-2, considered to be a benchmark oncogene and this result is counterintuitive, considering the degenerative character of the disease.

In another analysis, the control query [disease or disorder] and [activation or activator] generated ~125000 hits, while the query [disease or disorder] and [deactivate or deactivator or suppressor or repress or repressor] generated ~25000 hits. The target query [(Alzheimer’s or Alzheimer or neuropathy or neuropathic or neuro-degeneration or neurodegeneration or neurodegenerative or dementia)] and [activation or activator] produced 17500 hits, while the query [(Alzheimer’s or Alzheimer or neuropathy or neuropathic or neuro-degeneration or neurodegeneration or neurodegenerative or dementia)] and [deactivate or deactivator or suppressor or repress or repressor] produced ~1300 hits. The ratios point to neuropathies being more preferentially associated with activation processes (compare 125000:25000 for the control and 17500:1300 for the neuropathies).

Thus, we conclude that an analysis of entire PubMed shows that the neuropathy-related information is more closely and paradoxically associated with oncogenes and activation than with tumor suppressors and deactivation, confirming the trend observed in our data.

### A study of intersection for Alzheimer’s Disease and Angiotensin receptor blocker response pathways

The limited number of mechanistically relevant members comprising CDM list allows aligning with the literature data covering downstream effects of Angiotensin receptor AT-1. Both the results in Table 2 of the current study and the AT-1 literature review indicate AT-1 related genes as likely to mediate the effect of ARBs (angiotensin receptor blockers) on Alzheimer’s development.

Literature analysis points to significant interaction of AT-1 pathway with oncogene activation as well as with luteinizing hormone and insulin dependent pathways in neurons [30-35]. The role of oncogene modulation in response to AT1R blockers is complex, with some oncogenes being inhibited [30,31], while some being up-regulated [32]. In the cases when the ARBs exhibit neuroprotective effects via c-JUN inhibition, levels of other oncogenes remain as they were and the overall impact of oncogenic activation could still be executed through collateral routes. An example of such collateral pathway is a compensatory increase in activity of oncogenic angiotensin II receptor II (AT-2)/MAS pathway after the blockade of angiotensin II receptor I (AT-1) [36]. In mouse model, the alleviation of Alzheimer’s disease was experimentally achieved by hippocampal delivery of the oncogenic fibroblast growth factor FGF2 [37]. The predominance of neuroprotective effect in oncogene stimulation by ARBs is emphasized by ARB induction of IGF1, a molecule with a powerful anabolic and pro-survival impact [35]. Thus, the connection between AT1R inhibition and general activation of neuronal oncogenes is rather prominent in the body of research literature.

The LH/FSH regulation was previously linked to Alzheimer-like degeneration in murine models [38], thus, lending greater significance to stimulation of luteinizing hormone expression by angiotensin II that was previously observed in neurons [34].

Another group of entries in the higher score sub-networks 1 and 2 belongs to cytoskeleton rearrangement pathways. Cytoskeleton rearrangement related signaling that is the necessary step in vasoconstriction and vasodilation, a major short term effect of any anti-hypertensive drug. Angiotensin pathway is certainly involved in the pathways featured in the sub-networks 1 and 2 [39-41]. According to our data, the most abundant sub-networks of the Tier 1/sub-network 1 are the regulators of G-protein signaling and G-proteins: GNAS, GNB5, GNG3, GPRASP2, RGS4, RGS6, RGS7. The vascular remodeling and vasodilating role of GRS2, GRS3, GRS5, GRS18 and GNB3 in regulating of vascular tonicity in the context of Angiotensin receptor (AT-1) signaling was described previously in [42-46]. Vascular tone is maintained by the cytoskeleton rearrangement and the intracellular motility, thus connecting it to the protein misfolding and/or defective chaperone complex formation.

To conclude, substantial literature evidence connects the CDM derived in the current report and organized in the network of Table 2 with Angiotensin Receptor I pathway or its blockers, in the neuronal setting.

## Discussion

Inferring clinically relevant insights from the complex picture of the quantitative changes in gene expression levels remains a major challenge of systems biology. An interpretation of the disease signature remains the least standardized part of analytic procedures. In most cases, this analysis is riddled with subjective inference about whether given change in expression levels should be classified as causal, passively associated with observed phenotype or simply incidental to study design. Recent introduction of knowledge-based algorithms is expected to aid in producing reasonable hypotheses linking altered pathways to phenotypic changes. In this study, we attempted to devise knowledge-based algorithm capable of generating network clustering-validated, highly prioritized shortlists of potential targets pertinent to pathogenesis, the Compact Disease Models, or CDMs. To generate CDMs, we assume that molecular targets pertinent to pathogenesis of certain chronic disease may be recognized by their consistent visibility (differential expression, association of SNPs, functional evidence etc.) across most of independently designed experiments. In other words, a molecular target highlighted in a majority of studies (high-prevalence target) is more likely to be mechanistically important than the target detected in a minority of studies (low-prevalence target), although this relationship is may be not so straightforward [17]. The genes prioritized by relatively simple approach described in our study are later validated by assessing the reproducibility of the findings across other technological platforms. Only the strongest signals prevalent in all sample subsets (“compartments”) would pass such a rigorous filter. We believe that cross-validated, highly prevalent molecules are more likely to reflect more fundamental aspects of the disease and stand closer to causation. If the high-prevalence targets also form a highly clustered network, this association cross-validates substantial role for each of its constituents in the disease etiology, by compounding the difference in the probability of being truly causative between more and less prevalent signals.

In this report, Alzheimer’s Compact Disease Model (CDM) was built using both Illumina and Affymetrix platforms through extraction of differential expression data followed by tiering the gene expression evidence by its consistency. Both microarray platforms rely on oligonucleotide multi-probe approach; however, the experimental workflow, probe length, probe choice and signal processing statistics between the two platforms substantially differ [47,48]. In our approach, this inter-platform discrepancy is expected to serve as a filter that eliminates the signals that display poor consistency due to low reproducibility of expression level changes or due to elevated person-to-person expression variability, thus, cutting out the probability to detect meaningless (i.e. false positive) signals.

### Entropic disease model and pleiotropic role of angiotensin receptor blockers

Based on the observations of the current report, a pleiotropic model of early-stage Alzheimer disease could be proposed.

From very general considerations, a rigid differentiation program and complex shape of the neuron makes it an inherently disadvantaged cell type. Thermodynamically, expression of a gene in the nucleus that is followed by long route of the delivery of resultant protein to the target site at the synaptic junction either in a folded or properly pre-folded state is unfavorable due to high Boltzmann entropy loss associated with long processes. In the neurons, the travel distances may reach 0.5 -1 m; the maintenance of properly folded protein requires costly coordination of its intracellular traffic with the chaperone assembly sites and migration of the chaperone-protein complex to the destination. The high Boltzmann entropy loss of the process has to be matched by a high influx of free energy in the protein traffic path, derived in sufficient stimulation of anabolic and trophic pathways. This fundamental understanding is in agreement with our findings that the pathways jointly implicated in both blood pressure control and neurodegeneration are mostly anabolic and pro-survival. When anabolic pathways become down-regulated in CNS due to aging, neurotoxicity or mutation, it takes its toll on energy balance within the cell and increases the risk of misfolding. Thus, the long-term sustainability of anabolic processes in the neurons may be favored by regular anti-hypertensive treatments that assist cell survival.

In this balance of energy, the state of cytoskeleton organization determines the Boltzmann entropy loss. Disorganized cytoskeleton has higher initial entropy and the required intra-neuronal coordination would impose higher entropy costs. Thus, the intensity of anabolic processes is not the only factor determining energy supply for proper protein folding and trafficking. The luteinizing and follicle stimulating hormones (LH and FSH) both regulate menstrual cycle in females and spermatogenesis in males serving as upstream stimulators of androgen and estrogen production. Importantly, gonadotropins were found to be involved in the earliest stages of Alzheimer’s disease and in memory-related processes in humans and in multiple murine models [49,50]. Non-pituitary expression of FSH and its co-localization with FSH receptor and GnRH receptor in rat cerebellar cortex was shown previously [51]. Brain regions susceptible to degeneration in AD are enriched in both LH and its receptor; moreover, in animal models of AD, pharmacologic suppression of LH and FSH reduced plaque formation [52]. As both the oocytes meiosis that is triggered by LH and the process of spermatogenesis that is initiated by FSH require extensive cytoskeleton remodeling [53,54], it is tempting to speculate that the Boltzmann entropy state of neuronal cytoskeleton may be, in part, dependent on FSH and LH stimulation, possibly through G-protein activation. Respectively, G-protein regulators form a tightly connected cluster around FSH and LH nodes (Figure 3).

It is generally accepted that the probability of any chronic disease of old age increases in parallel with an increase in genomic entropy that degrades the complexity of epigenetic landscapes [55]. Age-dependent demethylation of the genome leads to an increase in the transcription of non-coding RNAs, while CpG-rich 5’ regions of select genes may become hypermethylated [56]. In case of neurons, the global hypomehylation and site-specific hypermethylation was found to be associated with degenerative and psychotic diseases [57,58]. In agreement with these observations, our data point to an overall decrease in transcript expression levels in the most of the functional categories showing high enrichment coefficients by GO-MINER. In some form, the down-regulation bias was traced among ~200 members of the Tiers 0—2 and ~1300 members of consistency Tier 3 and PCS groups. It is possible that this phenomenon is reflected by negative downstream changes in the stability of RNA transcripts and proteins, efficiency of translation and posttranslational modifications and, again, protein folding and trafficking. An exception to this trend is prominent up-regulation of NF-kB pathway (Figure 3, NF1C), that is involved in inflammation, cellular stress and apoptosis.

Another chromatin remodeling associated pathway is insulin signaling (Table 2). Importantly, IGF1 pathway is implicated in both life-span control and antihypertensive response. For example, a protective hormone Klotho, a competitive antagonist of IGF1 in kidney, is known to reverse degenerative nephropathies in murine models, and, as well, shown as downregulated in aging primates through chromatin methylation [59]. Interestingly, an inhibition of angiotensin II signaling by counteracting expression of IGF-II receptor is also shown to up-regulate Klotho [60,61]. Taken together, these data suggest a potential of antihypertensive agents to oppose the long-term age-related chromatin remodeling.

### Literature validation of the entropic model built based on CDM filtered gene list

The principle assumption of our study is that the pathways that relevant to the disease mechanism should be consistently discovered in a number of independent studies. Many pathways highlighted by our enrichment strategy were also described as experimental findings relevant to the context of early stages of Alzheimer’s disease, or, in general, the process of neurodegeneration. Extreme functional enrichment for protein traffic vesicle proteins observed in the CDM dataset points to a substantial role of this mechanism in the neurodegeneration, and is likely to be an early pathogenetic event. Some experimental reports confirm impairment of protein vesicle traffic in early stages of neurodegeneration [62,63]. The body of literature that discusses protein traffic vesicles in the context of neurodegeneration is relatively small and recent, as compared to more common and more general discussions of cytoskeleton and heat shock protein involvement in Alzheimer’s disease. Hence, we may conclude that the proposed CDM building technique aids the acquisition of relatively novel mechanistic insights underrepresented in broader literature.

Another important finding of the report is abundance of oncogenes in the Alzheimer’s disease interaction network built around the CDM gene list cut-off at a Tier 1 consistency. The independent literature search uncovers numerous publications describing the connection of oncogenes to improper, but possibly compensatory reactivation of cell cycle in terminally differentiated neurons that eventually leads to a cell death [64-66]. An alternative hypothesis points to the fact that patients with Alzheimer's disease have lower risk of incident cancer than general population [64,67,68]. One explanation to that paradox is a mitochondrial disfunction that is both implicated in early stages of Alzheimer’s disease development and impacted by the oncogene-tumor suppressor balance [66-68]. The connection between oncogene activation and bioenergy available to a neuron appears to be well described in the literature, in agreement with the conclusions of CDM-based analysis. The mechanistic support to the bioenergetic view of oncogene role over improper re-activation of cell cycle is provided by much higher score rank of the Ingenuity sub-network 2 (Table 2) comprising mitochondrial ATP-ase subunits vs. sub-networks 8 and 9, comprising cell cycle components.

Additionally, the CDM gene list analysis uncovers the prominent role of follicule-stimulating hormone, luteinizing hormone and gonadotropin in the development of early Alzheimer’s. The independent literature search presents evidence of increased expression of LH in the neurons vulnerable to Alzheimer’s disease [69]. In aged transgenic mice with Alzheimer-type of brain degeneration (Tg 2576), an ablation of the luteinizing hormone by a gonadotropin-releasing hormone analogue leuprolide acetate significantly attenuated cognitive decline and decreased amyloid-beta deposition as compared to placebo-treated animals [52]. Hence, the data presented in [69] and [52] and supported by CDM model indicate an involvement of FSH/LH pathway in Alzheimer’s.

The gene list distilling steps and its subsequent compacting are crucial to CDM-based hypothesis generation. If these steps would be omitted, the resultant CDM would be represented by impractically large gene network, dominated by the non-specific pathways common for many pathologies. In Alzheimers, non-compacted gene lists are dominated by stress response and inflammatory pathways marked as the highest scoring sub-networks. Without denying the aggravating role of inflammation in Alzheimer’s disease, inflammation abating approaches are unlikely to produce sustainable therapeutic results as they target relatively late stages of pathogenesis. Importantly, the distillation of the gene list into compact model (CDM) introduces an opportunity to catch a glimpse at possibly causative mechanistic alternatives that otherwise would took years to uncover through hypothesis-driven experimental studies that tend to look after overall plausibility of possible findings at the stage of the study design. While some models caution us against too stringent cutoffs for initial CDM composition [17], milder cutoffs that are combined with a cross-platform analysis appear to be a promising direction that requires further efforts.

## Conclusions

Here we present a novel knowledge-based algorithm that generates network clustering-validated, highly prioritized shortlists of potential targets pertinent to pathogenesis, the Compact Disease Models, or CDMs. This algorithm allowed us to generate a distilled, tiered list of Alzheimer’s disease-related genes and to derive a pleiotropic, network-based model for early stages of this disease. In this model, the first degree network associates were characterized by strong predominance of oncogenes. Loss of anabolic stimulation in neurons appears to progress with age due to promoter methylation, until the available free energy in the terminally differentiated cells would cease to compensate Boltzmann entropy loss that is due to the toll of the folding and long-distance delivery of the neuronal proteins. The prophylactic, anti-Alzheimer effect of the ARBs and beta blockers suggest that they play a role at the inception steps in the development of degenerative symptoms. Consequently, understanding of the pathways opposed by these agents has a substantial value since these pathways are likely to be causative to the degenerative process. Based on this logic, protein traffic vesicles, oncogenes, gonadotropin hormones and insulin-related pathway were identified as potential players in early Alzheimer’s disease. This understanding may aid in shifting the therapeutic efforts to the reversible stages of neurodegenerative disease, when the neuronal damage is relatively mild and self-perpetuating misfolded protein oligomers are not yet formed.

### Availability of support data

The data set(s) supporting the results of this article are included within the article and its Additional file 1.

## Abbreviations

ARBs: Angiotensin receptor blockers; AT-1: AT1R – Angiotensin receptor 1; CDM: Compact disease models.

## Competing interests

The authors declare that they have no competing interests.

## Authors’ contributions

Both authors contributed to the study design, interpretation of results and producing the manuscript. All authors read and approved the final manuscript.

## Supplementary Material

Additional file 1 **Datasets A, B and C: (the primary data).** Dataset D: Tiered consistency scores for all scored genes. Click here for file

Additional file 2 The outputs of GO-MINER ontological analysis of the genes within Consistency Tier 0. Click here for file

Additional file 3 The outputs of GO-MINER ontological analysis of the genes within Consistency Tier 1. Click here for file

Additional file 4 The outputs of GO-MINER ontological analysis of the genes within Consistency Tier 2. Click here for file

Additional file 5 The outputs of GO-MINER ontological analysis of the genes within Consistency Tier 3. Click here for file

Additional file 6 The sub-network composition for the Tier 1, including both experimental and inferred members. Click here for file

Additional file 7 The sub-network composition for the Tiers 1-3, including both experimental and inferred members. Click here for file
